# Diagnostic and Prognostic Value of Donor-Derived Cell-Free DNA in Acute Rejection After Kidney Transplantation: A Narrative Review

**DOI:** 10.3390/diagnostics16050668

**Published:** 2026-02-26

**Authors:** Stella Vasileiadou, Nikolaos Antoniadis, Asimina Fylaktou, Stavros Neiros, Filippos F. Karageorgos, Maria Stangou, Emmanouil Sinakos, Serafeim-Chrysovalantis Kotoulas, Eleni Massa, Eleni Mouloudi, Georgios Tsoulfas

**Affiliations:** 1 Department of Transplantation Surgery, Aristotle University of Thessaloniki, Hippokration General Hospital, 54642 Thessaloniki, Greecefilipposk@auth.gr (F.F.K.);; 2 Department of Immunology, National Peripheral Histocompatibility Center, Hippokration General Hospital, 54642 Thessaloniki, Greece; 3 1st Department of Nephrology, Aristotle University of Thessaloniki, Hippokration General Hospital, 54642 Thessaloniki, Greece; 4 4th Department of Internal Medicine, Aristotle University of Thessaloniki, Hippokration General Hospital, 54642 Thessaloniki, Greece; em_sinakos@yahoo.com; 5 Intensive Care Unit, Hippokration General Hospital, 54642 Thessaloniki, Greece

**Keywords:** kidney transplantation, donor-derived cell-free DNA, acute rejection, biomarkers, narrative review

## Abstract

**Background:** Kidney transplantation is the optimal treatment for end-stage renal disease; however, acute rejection remains a major determinant of long-term graft dysfunction and failure. Donor-derived cell-free DNA (dd-cfDNA) has emerged as a minimally invasive biomarker reflecting allograft injury, with growing evidence supporting diagnostic and prognostic utility. **Objectives:** This structured narrative review aimed to synthesize contemporary evidence (2020–2025) on the diagnostic and prognostic utility of plasma dd-cfDNA and its integration into kidney transplant rejection surveillance. **Methods**: A narrative literature review was conducted using PubMed to identify studies published or available online ahead of print, between January 2020 and September 2025, evaluating plasma dd-cfDNA in adult kidney transplant recipients. Manual screening of reference lists supplemented the search. Original clinical studies reporting diagnostic or prognostic outcomes were included, and the results were synthesized narratively due to methodological heterogeneity. **Results**: Across prospective and retrospective cohorts, elevated dd-cfDNA discriminated rejection from non-rejection biopsies, with strongest performance in antibody-mediated and microvascular rejection phenotypes. Longitudinal studies demonstrated that dd-cfDNA elevations often preceded histologically confirmed rejection and predicted adverse graft outcomes, while low levels were associated with immunologic quiescence. Assay variability limited cross-study comparability, whereas integration with donor-specific antibodies, gene expression profiling, or algorithm-based approaches improved diagnostic and prognostic discrimination. **Conclusions**: Dd-cfDNA is a clinically meaningful biomarker for kidney transplant rejection monitoring, providing diagnostic and prognostic information beyond conventional functional markers. When interpreted longitudinally and in clinical context, dd-cfDNA supports risk stratification and surveillance, with evidence supporting its expanding role in risk-adapted transplant care.

## 1. Introduction

Kidney transplantation remains the treatment of choice for patients with end-stage renal disease, offering superior survival, quality of life, and cost-effectiveness compared with long-term dialysis therapy [1]. Despite substantial advances in immunosuppression and peri-transplant care, acute rejection—defined as an immune-mediated injury to the transplanted kidney—continues to affect a significant proportion of kidney transplant recipients. Particularly, within the first year after transplantation, the incidence remains approximately 10–15% and represents a major contributor to chronic allograft dysfunction and premature graft loss [2]. Contemporary registry data indicate that subclinical inflammation and rejection, referring to histologic evidence of immune injury in the absence of overt clinical symptoms, are also associated with inferior long-term graft survival, highlighting the need for improved strategies for early detection and risk stratification [2,3].

From a pathophysiological perspective, acute rejection represents a complex interplay between alloimmune activation and structural injury to the transplanted kidney. Antibody-mediated rejection (ABMR) is characterized by donor-specific antibody (DSA) binding, complement activation, and microvascular inflammation, while T cell–mediated rejection (TCMR) reflects cellular infiltration and tubulointerstitial damage [4]. Importantly, these immunologic processes may precede detectable changes in serum creatinine or estimated glomerular filtration rate (eGFR), limiting the sensitivity of conventional functional biomarkers and often delaying diagnosis until significant tissue injury has occurred [5,6].

In current clinical practice, kidney allograft monitoring relies primarily on serum creatinine, proteinuria, and indication-based biopsies, with protocol biopsies reserved for selected centers. However, these approaches are invasive, resource-intensive, and poorly suited for longitudinal surveillance, particularly in stable patients or those managed in community-based transplant follow-up programs [7]. From a clinical and assistive care perspective, transplant recipients require coordinated, multidisciplinary monitoring involving transplant nephrologists, nursing specialists, laboratory services, and increasingly decentralized care models, highlighting the need for reliable, minimally invasive biomarkers that can be integrated into routine follow-up pathways [8].

Donor-derived cell-free DNA (dd-cfDNA) has emerged as a promising liquid biopsy biomarker, defined as short fragments of DNA released into the recipient’s bloodstream from injured donor kidney cells. Unlike immune activation markers, dd-cfDNA directly quantifies tissue damage and may therefore capture both overt and subclinical rejection processes [9,10].

Given the rapid expansion and increasing clinical uptake of dd-cfDNA assays over the last decade, together with persistent uncertainty regarding interpretation, threshold selection, and integration into practice, a focused appraisal of recent evidence (January 2020–September 2025) is timely and relevant for clinicians involved in kidney transplant care. The primary objective of this structured narrative review was to synthesize contemporary clinical evidence on the diagnostic performance of plasma dd-cfDNA for the detection of biopsy-proven acute rejection in adult kidney transplant recipients. The secondary objectives were to explore the broader clinical and prognostic implications of dd-cfDNA in kidney transplantation, to examine heterogeneity in its performance across clinical and histologic contexts, and to assess methodological and analytical factors influencing interpretation in routine practice.

Based on these objectives, the following research questions were formulated:

1. What is the diagnostic accuracy of plasma dd-cfDNA for identifying biopsy-proven acute rejection in adult kidney transplant recipients?
2. How does dd-cfDNA perform across rejection phenotypes?
3. Does dd-cfDNA provide prognostic information for future rejection events, de novo donor-specific antibody development, and longitudinal allograft function?
4. How do differences in assay platforms, analytical approaches, and applied thresholds influence dd-cfDNA interpretation?
5. What is the added diagnostic and clinical value of integrating dd-cfDNA with complementary biomarkers and algorithm-based approaches?

## 2. Materials and Methods

### 2.1. Literature Search Strategy

A structured narrative literature search was conducted in the PubMed database to identify relevant studies evaluating dd-cfDNA in kidney transplantation. The search combined free-text terms related to donor-derived cell-free DNA, kidney transplantation, acute rejection, and diagnostic or prognostic outcomes.

The search covered studies published between January 2020 and September 2025 and included articles that were available online ahead of print at the time of the search, corresponding to the contemporary clinical implementation era of plasma-based dd-cfDNA assays. The final search was performed on 30 September 2025.

The complete search strategy, including all search terms, Boolean operators, and stepwise query combinations, is detailed in Supplementary Table S1.

### 2.2. Eligibility Criteria

#### 2.2.1. Inclusion Criteria

Studies were eligible for inclusion if they met the following criteria:

• Original clinical research involving adult kidney transplant recipients (≥18 years);
• Quantitative assessment of plasma dd-cfDNA;
• Reported diagnostic and/or prognostic outcomes related to biopsy-proven acute rejection or allograft outcomes or described prospective dd-cfDNA–guided surveillance or diagnostic strategies with direct clinical relevance.

#### 2.2.2. Exclusion Criteria

Studies were excluded if they:

• Focused exclusively on pediatric kidney transplant recipients;
• Evaluated dd-cfDNA exclusively in urine samples;
• Were review articles, systematic reviews, meta-analyses, editorials, letters, or case reports without original clinical data.

Pediatric studies were excluded to reduce clinical heterogeneity related to immune maturation and pediatric-specific immunosuppression protocols. Urine-based dd-cfDNA studies were excluded because plasma-based assays currently represent the primary modality for analytical validation and routine clinical implementation in adult transplant practice.

### 2.3. Study Selection and Data Extraction

Titles and abstracts were screened for relevance, followed by full-text review of potentially eligible studies. Reference lists of relevant reviews and editorials were manually screened to identify additional primary studies.

From each included study, data were extracted on study design, population characteristics, sample size, dd-cfDNA assay platform and analytical method, applied threshold, sampling strategy, type of rejection, and reported diagnostic or prognostic outcomes.

### 2.4. Data Synthesis

Given the heterogeneity in study design, patient populations, assay platforms, and reported endpoints, findings were synthesized narratively.

### 2.5. Study Selection Results

The literature search identified 42 records, of which 21 studies (20 original clinical studies and one protocol study with direct clinical relevance) met eligibility criteria and were included in the final narrative synthesis. Additional publications were reviewed to provide contextual background, while studies focusing exclusively on pediatric populations or urine-based dd-cfDNA analyses were excluded.

## 3. Results

### 3.1. Study Characteristics

The reviewed literature included 21 studies evaluating plasma dd-cfDNA in kidney transplantation. Most studies focused on adult kidney transplant recipients, while a minority included mixed adult–pediatric populations. Study designs encompassed multicenter prospective cohorts, with or without external validation, large real-world registry-based observational studies, single-center diagnostic or observational studies, and randomized or algorithm-based diagnostic strategy studies.

Sample sizes varied widely, ranging from small single-center cohorts (*n* = 28) to large multicenter populations exceeding 3500 participants. Across studies, dd-cfDNA was quantified using several commercially available analytical platforms, predominantly single nucleotide polymorphism–based next-generation sequencing (SNP-based NGS), which distinguishes donor and recipient DNA by sequencing genetic variants unique to each individual. Fewer studies employing droplet digital polymerase chain reaction (ddPCR), a highly sensitive PCR-based method that enables absolute quantification of donor DNA fragments. Despite methodological differences, all platforms aimed to quantify either the fractional or absolute concentration of donor-derived DNA fragments in recipient plasma.

Sampling strategies differed according to study objectives and included dd-cfDNA measurement at the time of indication and/or protocol biopsy, during routine post-transplant surveillance, through longitudinally monitoring for prognostic assessment or strategy-driven sampling approaches. Rejection phenotypes evaluated across studies comprised ABMR, TCMR, mixed rejection, borderline changes (histologic findings suggestive but not fully diagnostic of acute rejection), and microvascular inflammation or injury (MVI, capillary-level inflammatory damage often associated with antibody activity), as defined by contemporary Banff criteria.

Key characteristics of the included studies—including study design, sample size, dd-cfDNA assay platform, sampling strategy and rejection phenotypes—are summarized in Table 1, providing a structured overview that facilitates qualitative comparison across heterogeneous study designs.

#### 3.1.1. Diagnostic Performance of dd-cfDNA at the Time of Biopsy

Dd-cfDNA levels were consistently higher in cases of biopsy-proven rejection compared with non-rejection biopsies. In the largest prospective multicenter study, Aubert et al. [22], evaluated 2882 kidney transplant recipients with paired dd-cfDNA samples and biopsies, including both for-cause and protocol biopsies. Dd-cfDNA was independently associated with the presence, activity and severity of rejection, including ABMR, TCMR and mixed phenotypes. Diagnostic performance improved when dd-cfDNA was analyzed as a continuous variable, while integration with standard clinical parameters increased the area under the curve (AUC) from 0.777 to 0.821. These findings were reproducible in an external validation cohort, supporting the robustness and calibration of the biomarker across centers.

Comparable results were reported across different analytical workflows. Loupy et al. [21] showed that decentralized dd-cfDNA testing demonstrated high concordance with centralized laboratory assays (r^2^ = 0.95), with similar AUC (~0.76) for detecting rejection. These findings support the analytical robustness and transferability of dd-cfDNA testing across laboratory settings.

Smaller observational studies further corroborated these results. Kim et al. [23] demonstrated that dd-cfDNA levels were higher at the time of indication biopsy in recipients with rejection compared with those without, using a threshold of 0.4%. Mantios et al. [20] reported that dd-cfDNA levels above 0.5% detected acute rejection more effectively than serum creatinine. Earlier studies, including Zhang et al. [11] reported high diagnostic accuracy for ABMR, with AUC values almost approaching 0.9 at thresholds around 1%. In contrast, a single-center study by Gielis et al. [6], found that although dd-cfDNA levels were higher during rejection episodes, the diagnostic performance of the biomarker was modest and comparable to serum creatinine.

Real-world registry data further support the diagnostic utility of dd-cfDNA. In the study by Bromberg et al. [27], biopsies performed in the setting of elevated dd-cfDNA had higher rejection yield than those with non-elevated levels, both for surveillance and for-cause biopsies. The overall AUC for biopsy-proven acute rejection was 0.79, indicating good discriminative performance in real-world clinical practice.

#### 3.1.2. Detection of Subclinical Rejection and Microvascular Injury

Several studies evaluated dd-cfDNA in clinically stable kidney transplant recipients undergoing protocol biopsies. In the large multicenter cohort by Aubert et al. [22], dd-cfDNA was independently associated with subclinical rejection in recipients with stable renal function, highlighting the ability of the biomarker to detect ongoing allograft injury despite unremarkable conventional monitoring.

In a post hoc analysis of the CTOT-08 study [14], conducted in stable recipients undergoing protocol biopsies, dd-cfDNA levels ≥ 0.7% identified subclinical ABMR more effectively than gene expression profiling, a blood-based transcriptomic assay reflecting immune activation. Importantly, when dd-cfDNA was combined with gene expression profiling, overall diagnostic performance improved, with the AUC reaching approximately 0.81 and a high negative predictive value (88%) when both biomarkers were negative.

Kim et al. [23] extended these observations by focusing on MVI. In this study, dd-cfDNA levels were elevated in DSA-negative recipients with severe MVI and outperformed both DSA testing and serum creatinine. Furthermore, dd-cfDNA correlated quantitatively with MVI scores, and its combination with DSA improved the detection of ABMR. Similarly, Sellarés et al. [24] reported that dd-cfDNA was strongly associated with ABMR, mixed rejection, and MVI—including subclinical phenotypes—whereas gene expression profiling alone showed limited correlation with histologic injury.

#### 3.1.3. Temporal Dynamics and Early Prediction of Rejection

Beyond cross-sectional diagnostic performance, several studies demonstrated that elevations in dd-cfDNA may precede histologically confirmed rejection. In a registry-based analysis, Bromberg et al. [10] reported that dd-cfDNA levels increased months before biopsy-proven rejection, with a median lead time of approximately five months for ABMR and two months for TCMR. In contrast, serum creatinine did not predict impending rejection. Moreover, repeated dd-cfDNA elevations in the absence of biopsy-proven rejection were associated with lower eGFR, suggesting ongoing subclinical allograft injury.

Consistent with these observations, the ADMIRAL study [18], a large prospective multicenter cohort including more than 1000 recipients followed for up to three years, demonstrated that persistently elevated dd-cfDNA levels (≥0.5%) were associated with subsequent rejection events, development of de novo donor-specific antibodies (dnDSA), and future decline in eGFR. Conversely, stable low dd-cfDNA levels were associated with immunologic quiescence and more favorable graft trajectories.

Additional longitudinal surveillance studies further support the dynamic nature of dd-cfDNA. Shen et al. [13] reported a rapid decline in dd-cfDNA levels following anti-rejection therapy, which correlated with renal functional recovery over subsequent months, highlighting the potential utility of dd-cfDNA for monitoring treatment response.

Finally, a randomized diagnostic strategy trial in kidney transplant recipients with dnDSA demonstrated that a dd-cfDNA–guided biopsy approach enabled earlier diagnosis of active or chronic ABMR compared with standard clinically guided biopsy strategies [29].

#### 3.1.4. Prognostic Associations with Graft Outcomes

In addition to early rejection prediction, evidence from multiple cohorts supports prognostic associations between dd-cfDNA levels and graft outcomes, encompassing both baseline measurements and longitudinal dynamics.

In the ADMIRAL cohort [18], persistently elevated dd-cfDNA levels were associated with an approximately threefold increased risk of developing dnDSA and an almost twofold increased risk of subsequent eGFR decline. Importantly, these associations remained significant after adjustment for clinical covariates, supporting dd-cfDNA as an independent prognostic biomarker.

Similarly, Stites et al. [12] evaluated kidney transplant recipients with early TCMR 1A or borderline rejection and demonstrated that dd-cfDNA ≥0.5% identified patients at increased risk for persistent or recurrent rejection, dnDSA formation, and deterioration of renal function. Notably, dd-cfDNA enabled additional risk stratification within histologically heterogeneous early rejection phenotypes, complementing conventional Banff classification.

Beyond baseline risk stratification, several studies explored dd-cfDNA kinetics as markers of treatment response and short-term prognosis. In a prospective longitudinal cohort, Shen et al. [13] reported a rapid decline in dd-cfDNA levels following anti-rejection therapy, with changes in dd-cfDNA correlating with renal functional recovery at 1, 3, and 6 months. These findings highlight dd-cfDNA as a dynamic biomarker reflecting therapeutic response rather than a static diagnostic indicator.

Further supporting the prognostic relevance of longitudinal trends, Bunnapradist et al. [28] reported that unfavorable dd-cfDNA trajectories following biopsy-proven rejection were associated with adverse composite outcomes at 12 months, including recurrent rejection, dnDSA development, persistent graft dysfunction, or graft loss. These results emphasize the superior prognostic value of serial dd-cfDNA measurements compared with isolated single time-point assessments.

Consistent observations were also reported in smaller cohorts. Mantios et al. [20] found that dd-cfDNA levels > 0.5% at the time of rejection were associated with non-improving eGFR at one year, whereas decreasing dd-cfDNA levels after treatment characterized responders.

Finally, long-term prognostic implications of routine dd-cfDNA surveillance are being investigated in large prospective studies. The TRACK study [15] is a phase IV observational protocol evaluating dd-cfDNA–guided monitoring in relation to allograft survival, graft function decline, and dnDSA development; outcome data are currently pending.

#### 3.1.5. Combined Biomarkers Strategies

Evidence from multiple studies indicates that integrating dd-cfDNA with complementary biomarkers improves diagnostic performance beyond single-marker approaches. Park et al. [14] demonstrated complementary diagnostic roles of dd-cfDNA and gene expression profiling for subclinical rejection: dd-cfDNA showed superior performance for detecting ABMR, whereas gene expression profiling was more effective for identifying TCMR. Integration of continuous scores from both assays resulted in improved overall discrimination for subclinical rejection.

Ιn a real-world multicenter setting, Sellares et al. [24] evaluated dd-cfDNA in combination with DSA and gene expression profiling. In this study, dd-cfDNA demonstrated the strongest association with ABMR and MVI, while diagnostic performance was further improved when dd-cfDNA was combined with DSA.

Similarly, Yu et al. [25] assessed the combined use of dd-cfDNA and CXCL-10, an interferon-inducible chemokine associated with T-cell–mediated inflammation and allograft rejection, and reported improved diagnostic accuracy compared with either biomarker alone, with both markers independently associated with acute rejection. In another study, Akalin et al. [16] demonstrated that integrating dd-cfDNA with a five-gene immune quiescence signature (a transcriptomic profile indicative of low immune activation and stable graft function) significantly increased the AUC for distinguishing rejection from immune quiescence.

Beyond biomarker combinations, algorithm-based approaches incorporating different quantitative aspects of dd-cfDNA were associated with higher diagnostic performance. Halloran et al. [19] reported improved sensitivity, specificity, and overall diagnostic accuracy using a two-threshold algorithm that combined dd-cfDNA fraction and absolute quantity. Comparable findings were reported by Nie et al. [26], who applied a similar fraction–quantity strategy and demonstrated improved predictive performance using double-positive and double-negative classification approaches.

Alternative analytical strategies were also reported. Verhoeven et al. [17] validated a dd PCR–based method for absolute dd-cfDNA quantification and demonstrated significantly higher dd-cfDNA copy numbers during acute rejection compared with non-rejection.

## 4. Discussion

Although most available evidence derives from observational cohorts, the consistency of findings across large multicenter studies, real-world registries, and longitudinal designs supports donor-derived cell-free DNA as a clinically relevant non-invasive biomarker providing diagnostic and prognostic information beyond conventional functional markers in kidney transplantation [18,22,27].

### 4.1. Diagnostic Role of dd-cfDNA

With regard to the first research question, available evidence from the DART trial and subsequent multicenter cohorts demonstrates that plasma dd-cfDNA discriminates biopsy-proven acute rejection from non-rejection at the time of biopsy [9,18,30,31]. However, because dd-cfDNA reflects allograft injury rather than immune activation, its diagnostic performance is inherently context-dependent and lacks absolute specificity for rejection. The improved performance observed when dd-cfDNA is analyzed as a continuous variable underscores that rejection represents a biologically continuous process, best addressed through probabilistic interpretation rather than rigid cut-off–based decision-making [9,12,32].

Addressing the second research question, differences in performance across rejection phenotypes indicate that dd-cfDNA sensitivity scales with the burden and microvascular distribution of graft injury, rather than with rejection phenotype classification alone [18,31]. In addition, its ability to detect subclinical rejection and microvascular injury extends its diagnostic relevance beyond overt graft dysfunction in clinically stable recipients [23,30,31].

### 4.2. Prognostic Role of dd-cfDNA

Converging evidence supports dd-cfDNA as a prognostic biomarker, with sustained or rising levels frequently preceding histologically confirmed rejection by weeks to months [10]. Beyond early rejection prediction, persistently elevated dd-cfDNA levels independently predict adverse graft-related outcomes, including dnDSA development, functional decline, and recurrent rejection, even after adjustment for clinical covariates [18]. Importantly, longitudinal dd-cfDNA trajectories provide incremental prognostic information beyond conventional Banff classification, enabling identification of high-risk patients and assessment of treatment response despite similar histologic findings [12,13,28].

### 4.3. Interpretation of dd-cfDNA Cut-Off Variability

Differences in assay platforms, analytical approaches, and applied thresholds directly influence dd-cfDNA interpretation by limiting the clinical validity of fixed cut-off values across settings. Clinical context further constrains threshold-based interpretation, as early post-transplant elevations related to ischemia–reperfusion injury, along with sensitization status, retransplantation, and concurrent graft injury, introduce variability that cannot be adequately captured by a single universal cut-off [30,32,33].

Taken together, these considerations argue against isolated threshold-based interpretation and support a longitudinal, context-dependent approach to dd-cfDNA assessment that prioritizes continuous analysis and intra-patient trends over absolute cut-off values [32,33].

### 4.4. Multimodal Biomarker Integration and Algorithm-Based Approaches

Growing evidence indicates that integrating dd-cfDNA with complementary molecular and immunologic biomarkers provides incremental diagnostic and clinical value beyond single-marker approaches [14,23,24,34]. In this context, dd-cfDNA functions as a central marker of graft injury, while integration with gene expression profiling or DSA enhances discrimination between rejection phenotypes and improves specificity and negative predictive value across clinical settings [30,32,33].

Algorithm-based strategies further extend the clinical utility of dd-cfDNA by integrating complementary quantitative dimensions, such as fractional and absolute measurements, thereby supporting probability-based rather than binary diagnostic frameworks [19].

### 4.5. Perspectives for Clinical and Assistive Practice

From a clinical perspective, dd-cfDNA should be viewed as a complementary tool to optimize the timing and indication of kidney allograft biopsy rather than as a replacement for histologic assessment [32,33,35]. In surveillance settings, persistently low dd-cfDNA levels may support deferral of invasive testing, whereas rising or persistently elevated values can justify earlier biopsy or intensified monitoring [7,8,10]; in for-cause scenarios, dd-cfDNA refines pre-test probability and helps prioritize patients most likely to benefit from histologic evaluation [22,31]. Integrated dd-cfDNA–guided surveillance strategies may therefore support a transition toward continuous, risk-adapted monitoring, reducing unnecessary biopsies and enabling earlier detection of subclinical injury [14,18,34].

### 4.6. Limitations and Future Directions

Despite the expanding evidence base, several limitations warrant consideration. Most available data derive from observational studies, while randomized trials evaluating dd-cfDNA–guided clinical decision-making remain limited [33,36]. In addition, non-rejection causes of dd-cfDNA elevation—including infection, ischemia–reperfusion injury, and recurrent or de novo kidney disease—require further clarification [7,32], while long-term outcome data on chronic allograft rejection and graft dysfunction remain limited [29].

Future research should prioritize prospective validation of multimodal diagnostic and prognostic algorithms, standardization of pre-analytical and analytical procedures, and formal cost-effectiveness analyses [34]. Long-term surveillance studies will be essential to define the role of dd-cfDNA in graft survival and its integration into risk-adapted post-transplant care.

## 5. Conclusions

Donor-derived cell-free DNA (dd-cfDNA) has emerged as a clinically meaningful non-invasive biomarker with both diagnostic and prognostic relevance in kidney transplantation. Current evidence demonstrates reliable discrimination between rejection and non-rejection, with particularly strong performance in antibody-mediated and microvascular injury phenotypes, and indicates that dd-cfDNA elevations frequently precede clinical or functional deterioration.

Increasingly, dd-cfDNA is best interpreted as a dynamic marker of ongoing allograft injury rather than through isolated cut-off values. Its greatest clinical value lies in longitudinal assessment and in multimodal, algorithm-based strategies integrating dd-cfDNA with immunological and clinical parameters. Despite remaining challenges related to assay heterogeneity and standardization, dd-cfDNA already offers clinically actionable insights and is likely to play an expanding role in risk-adapted surveillance strategies in kidney transplant care.

## Figures and Tables

**Table 1:** Key characteristics of original clinical studies evaluating plasma dd-cfDNA in kidney transplantation.

Author	Journal (Year)	Study Design	Kidney Transplant Recipients (*n*)	Dd-cfDNA Assay Platform	Sampling Strategy	Rejection Phenotypes
Zhang et al. [11]	Frontiers in Immunology (2020)	Single-center prospective cohort	37	SNP-based NGS	Biopsy and surveillance	ABMR, borderline
Gielis et al. [6]	Nephrology Dialysis Transplantation (2020)	Multicenter prospective cohort	107	SNP-based NGS (Sequido)	Protocol biopsy, indication biopsy and surveillance	ABMR, TCMR, mixed, borderline
Stites et al. [12]	American Journal of Transplantation (2020)	Multicenter prospective cohort	79	SNP-based NGS (AlloSure)	Protocol biopsy, indication biopsy	TCMR 1A, borderline
Shen et al. [13]	Clinical Transplantation (2020)	Single-center prospective longitudinal cohort	28	SNP-based NGS	During and after rejection treatment	ABMR, TCMR
Park et al. [14]	Clinical Journal of the American Society of Nephrology (2021)	Multicenter post hoc analysis of prospective cohort (CTOT-08)	208	SNP-based NGS (TRAC)	Protocol biopsy	Subclinical ABMR and TCMR
Dale et al. [15]	JMIR Research Protocol (2021)	Multicenter prospective longitudinal cohort; Study protocol (TRACK)	3500	SNP-based NGS (AlloSure)	Protocol biopsy, indication biopsy and surveillance	NA
Akalin et al. [16]	Kidney360 (2021)	Multicenter diagnostic validation cohort	191	SNP-based NGS (AlloSure)	Biopsy	ABMR, TCMR, mixed
Verhoeven et al. [17]	Transplantation (2022)	Single-center method validation diagnostic study	223	Droplet digital PCR	Indication biopsy and surveillance	Acute rejection
Bu et al. [18]	Clinical Investigation (ADMIRAL study) (2022)	Multicenter prospective longitudinal cohort	1092	SNP-based NGS (AlloSure)	Surveillance and indication testing	Clinical and subclinical rejection
Halloran et al. [19]	Transplantation (2022)	Multicenter diagnostic algorithm validation study (Trifecta study)	NA (367 biopsy -matched samples)	SNP-based NGS (Prospera)	Indication biopsy	ABMR, TCMR, mixed
Mantios et al. [20]	Transplantation International (2023)	Single-center prospective diagnostic cohort	62	SNP-based NGS (AlloSeq)	Indication biopsy and surveillance	ABMR, TCMR
Loupy et al. [21]	Transplantation International (2024)	Multicenter prospective diagnostic validation cohort	580	SNP-based NGS (AlloSeq/AlloSure)	Protocol biopsy, indication biopsy	ABMR, TCMR, mixed
Aubert et al. [22]	Nature Medicine (2024)	Multicenter prospective longitudinal cohort (with external validation)	2882	SNP-based NGS (AlloSeq/AlloSure)	Protocol biopsy, indication biopsy	ABMR, TCMR, mixed
Bromberg et al. [10]	Transplantation (2024)	Multicenter registry-based observational cohort	424	SNP-based NGS (Prospera)	Surveillance	ABMR, TCMR
Kim et al. [23]	Frontiers in Immunology (2024)	Single-center prospective diagnostic cohort	64	SNP-based NGS (AlloSeq)	Indication biopsy	ABMR, TCMR, mixed, MVI
Sellares et al. [24]	Transplantation (2025)	Multicenter cross-sectional diagnostic study	193	SNP-based NGS (TRAC)	Protocol biopsy, indication biopsy	ABMR, TCMR, mixed, borderline, MVI
Yu et al. [25]	Transplantation Immunology (2025)	Single-center case–control diagnostic study	31	NA	At acute rejection diagnosis	Acute rejection
Nie et al. [26]	Diagnostics (2025)	Single-center prospective diagnostic cohort	50	SNP-based NGS + qPCR-based absolute quantification	Indication biopsy	ABMR, TCMR, mixed
Bromberg et al. [27]	American Journal of Transplantation (2025)	Multicenter registry-based observational cohort (KOAR)	1743	SNP-based NGS (AlloSure)	Surveillance and indication testing	ABMR, TCMR, mixed, borderline
Bunnapradist et al. [28]	American Journal of Transplantation (2025)	Multicenter prospective prognostic cohort	66	SNP-based NGS (Prospera)	Post-rejection monitoring	ABMR, TCMR, mixed
Akifova et al. [29]	Nephrology Dialysis Transplantation (2025)	Single-center randomized diagnostic strategy trial	40	Droplet digital PCR	Monitoring with biopsy triggered by dd-cfDNA	ABMR

Abbreviations: ABMR, antibody-mediated rejection; dd-cfDNA, donor-derived cell-free DNA; MVI, microvascular injury; NA, not available or not applicable (as reported in the original studies); PCR, polymerase chain reaction; qPCR, quantitative polymerase chain reaction; SNP-based NGS, single nucleotide polymorphism-based next-generation sequencing; TCMR, T cell–mediated rejection.

## Data Availability

No new data were created or analyzed in this study. Data sharing is not applicable to this article.

## Supplementary Materials

The following supporting information can be downloaded at: https://www.mdpi.com/article/10.3390/diagnostics16050668/s1, Table S1: PubMed search strategy and stepwise query combinations (January 2020–September 2025).

## Abbreviations

The following abbreviations are used in this manuscript:

ABMR	Antibody-mediated rejection
AUC	Area under the curve
dd-cfDNA	Donor-derived cell-free DNA
ddPCR	Droplet digital PCR
dnDSA	De novo donor-specific antibodies
DSA	Donor-specific antibodies
eGFR	Estimated glomerular filtration rate
HR	Hazard ratio
KTR	Kidney transplant recipients
MVI	Microvascular inflammation/injury
NA	Not available or not applicable
PCR	Polymerase chain reaction
SNP-based NGS	Single nucleotide polymorphism-based next-generation sequencing
TCMR	T cell-mediated rejection
