# Supplementary material for: Diagnostic and Prognostic Value of Donor-Derived Cell-Free DNA in Acute Rejection After Kidney Transplantation: A Narrative Review
Source: Diagnostics (Basel). 2026 Feb 26;16(5):668. doi: 10.3390/diagnostics16050668 (PMC12984478; doi:10.3390/diagnostics16050668)
Supplement: Supplementary file 1 [file diagnostics-16-00668-s001.zip › diagnostics-4098189-supplementary.pdf]

Supplementary Table S1 PubMed search strategy and stepwise query combinations (January 2020 – September 2025).

Search number	Query	Sort By	Filters	Results
**1**	donor-derived cell-free DNA			506
**2**	donor derived cfDNA			449
**3**	dd-cfDNA			291
**4**	cell-free DNA			65,573
**5**	(((#1) OR (#2)) OR (#3)) OR (#4)			65,584
**6**	kidney transplantation			163,429
**7**	renal transplant			193,024
**8**	(#6) OR (#7)			193,024
**9**	acute rejection			32,792
**10**	allograft rejection			45,305
**11**	(#9) OR (#10)			63,373
**12**	diagnostic			12,614,026
**13**	diagnostic performance			2,236,525
**14**	prognostic			495,455
**15**	predict*			2,637,869
**16**	outcome			3,942,450
**17**	((((#12) OR (#13)) OR (#14)) OR (#15)) OR (#16)			15,456,054
**18**	(((#5) AND (#8)) AND (#11)) AND (#17)			206
**19**	(((#5) AND (#8)) AND (#11)) AND (#17)		from 2020/01/01-2025/09/30	166
